# How suicide-bereaved family members experience the inquest process: a qualitative study using thematic analysis

**DOI:** 10.1080/17482631.2018.1563430

**Published:** 2019-01-29

**Authors:** Ailbhe Spillane, Karen Matvienko-Sikar, Celine Larkin, Paul Corcoran, Ella Arensman

**Affiliations:** a School of Public Health, University College Cork, Cork, Ireland; b National Suicide Research Foundation, Cork, Ireland; c Department of Emergency Medicine, University of Massachusetts Medical School, Worcester, MA, USA

**Keywords:** Inquest, coroner, qualitative, suicide, bereavement, family members

## Abstract

**Purpose:** Suicide bereavement confers unique risk and distress. In several countries, bereaved family members are called on to attend an inquest, an official public inquiry into deaths caused by external factors. The current study aimed to explore how suicide-bereaved family members (n = 18) experienced the inquest process, through qualitative semi-structured interviews.

**Method:** Participants were identified via coroner’s records and had previously taken part in a case-control study.

**Results:** Qualitative findings indicated four overall themes with respect to family members’ experiences of the inquest process: “inquest as fearfully unknown”, “structural processes of the inquest”, “enduring public and private pain to obtain answers” and “gaining answers and making sense”. Most family members experienced distress and fear as a result of several elements of the inquest process. Some participants had positive experiences but these did not outweigh the distress experienced by the majority of family members regarding their overall experience of the inquest process.

**Conclusions:** Key recommendations include informing family members of the main aspects and purpose of the inquest process beforehand, adapting the process to maximise the privacy and comfort of the bereaved relatives, and restricting graphic evidence being heard, where possible, to minimise distress experienced by family members.

## Introduction

A suicide death, while an individual act, creates a “ripple effect” which profoundly impacts familial (Pitman, Osborn, King, & Erlangsen, 2014), social (Cerel, Jordan, & Duberstein, 2008) and societal networks. This ripple effect is best illustrated by the Circles of Vulnerability Model (Lahad & Cohen, 2004) and the Social-Ecological Model (Dahlberg & Krug, 2002). The Circles of Vulnerability Model is based on the idea that every suicide is akin to a stone being thrown into a pool of water—ripples spread outwards to the edge of the water. The ripple effects are larger closer to the point of impact. The extent of vulnerability to suicide can consist of geographical, social and psychological proximity to the deceased. In short, people who discover the body of a person who has died by suicide, people in close familial or social circles with the deceased, or those who felt psychologically close to the deceased represent particularly vulnerable people to the effects of suicide bereavement (Lahad & Cohen, 2004). This model and the Social-Ecological Model each have a number of interconnecting levels which overlap to signify how factors at one level impact those at the next level.

Meaning-making following loss is crucial, but can be especially complex for suicide-bereaved family members, (Currier, Holland, & Neimeyer, 2006; Pritchard & Buckle, 2017) as they struggle with mental, physical and psychosomatic problems, including depression, nausea, vomiting, diabetes and hypertension (Bolton et al., 2013; Spillane et al., 2017; Spillane, Matvienko-Sikar, Larkin, Corcoran, & Arensman, 2018). Family members bereaved by suicide are at an increased risk of mood disorders, substance use disorders, complicated grief and post-traumatic stress disorder (de Groot, de Keijser, & Neeleman, 2006; Erlangsen et al., 2017). Additionally, suicide-bereaved family members have a heightened risk of suicidal behaviour, including self-harm and suicide, with partners and parents having the most distinct risk (Agerbo, 2005; Agerbo, 2003; Pitman, 2018; Pitman et al., 2014). Feelings of guilt, shame, stigma and rejection are common (de Groot et al., 2006; Harwood, Hawton, Hope, & Jacoby, 2002; Pitman, Osborn, Rantell, & King, 2016; Pitman, Rantell, Marston, King, & Osborn, 2017; Pitman, Stevenson, Osborn, & King, 2018), which can disrupt the meaning-making process. Support is crucial for people bereaved by suicide, yet they are less likely to receive informal support than people bereaved by sudden natural or unnatural causes of death (Pitman et al., 2017). They are also less likely to receive immediate support and more likely to report a delay in receiving support than people bereaved by sudden natural causes of death (Pritchard & Buckle, 2017). Proactively seeking formal and informal support can also be hampered by physical and psychosomatic health manifestations of grief, including low energy, poor appetite and insomnia (Hoffmann, Myburgh, & Poggenpoel, 2010; McKinnon & Chonody, 2014). Previous research also indicates educational and employment functioning can be hampered due to lack of institutional support, poor concentration levels due to grief and socially withdrawing from others for fear of stigmatisation (Pitman, Khrisna Putri, et al., 2018).

Many current and previous countries of the British Commonwealth use the coronial system to investigate suicide deaths and other deaths by external causes, where public inquests are held. However, little qualitative research exploring how the inquest process impacts on family members has been conducted (Biddle, 2003; Chapple, Ziebland, & Hawton, 2012; McKinnon & Chonody, 2014), with most of the research conducted some 40 years ago (Barraclough & Shepherd, 1976, 1977; Shepherd & Barraclough, 1974). Reviews of the coroner service (Haskins, 2000) and services available for people bereaved by suicide (Petrus Consulting et al., 2008) have been conducted in Ireland, but the experiences of family members following the inquest process are absent from both.

Many aspects of the procedures after a suicide can be distressing for family members, including the timing and setting of the inquest, the presence of media, insensitive media reporting of the suicide and the perceived invasion of privacy (Biddle, 2003; McKinnon & Chonody, 2014). The inquest process could help to provide some answers and facilitate meaning-making for bereaved family members, but this may not occur due to its public and potentially stigmatising nature (McKinnon & Chonody, 2014). This is one of the few studies which explores peoples’ experiences of the inquest process following their family member’s suicide.

## Methods

### Theoretical approach

Social constructionism was the theoretical underpinning of this study. Social constructionism posits that knowledge is constructed rather than created and that people psychologically construct their experiences through a social rather than an individual focus (Andrews, 2012). Society and social processes are therefore seen as the underlying mechanisms for how people comprehend and interpret the world around them (Lyons & Coyle, 2016). The knowledge that an individual assumes is also inextricably linked to their exposure to social, historical or political processes (Lyons & Coyle, 2016). Therefore, knowledge is socially and culturally constructed through interaction with others. Similarly, individuals learn culturally appropriate responses to grief. One of these responses is engaging in a meaning-making process, where individuals are forced to reconstruct life and the world around them (Castelli Dransart, 2013; Dyregrov et al., 2011; Neimeyer, Klass, & Dennis, 2014). This reconstruction is inevitably tied to and influenced by our social world (Neimeyer et al., 2014). However, this meaning-making process may be thwarted for people bereaved by suicide, with some wanting to conceal the cause of death for fear of being stigmatised by the community. Research indicates that people bereaved by suicide report more stigma and experience higher levels of guilt, shame and responsibility than people bereaved by sudden natural and sudden unnatural causes of death (Pitman et al., 2016). Therefore, meaning-making following bereavement, especially suicide bereavement, does not occur in a vacuum and is shaped by one’s social world. It is therefore critical to be aware that one’s social reality may impact on how one reacts to a suicide bereavement, and consequently the inquest process. The latter point is critical given that potentially inappropriate aspects of the inquest process, as experienced by family members may further strengthen stigmatising responses to them and the suicide, resulting in further isolation from the community.

### Study design and setting

This is a qualitative study, which utilised semi-structured interviews with 18 people bereaved by a family member’s suicide. These data for this study were collected during interviews for a previously published mixed methods study (Spillane et al., 2017, 2018). The aim of the previous mixed methods study was to examine the physical and psychological health effects of suicide bereavement on family members in Ireland. From the outset, the inquest process was spoken about by participants as an important aspect of their experiences following their family member’s suicide. Given that the conduct and procedures underlying inquest processes has the potential to negatively impact on family members, it was deemed necessary to explore this phenomenon in more depth. Questions on the inquest process were incorporated into the topic guide in an iterative approach to data collection in the mixed methods study, while the content of these was guided by the commentary noted in the first few interviews conducted. Semi-structured interviews were selected as the most appropriate data collection methodology as it allows for flexibility to discuss issues that may arise during the interview that require further probing. Since this area is understudied, semi-structured interviews provided the scope to explore family members’ experiences in an in-depth way, while also providing a rich description of the phenomena under investigation, in this case, family members’ experiences of suicide bereavement and the subsequent inquest process. Ethical approval was granted from the Clinical Research Ethics Committee of the Cork Teaching Hospitals.

### Sample and recruitment

Between 2014 and 2016, consecutive cases of suicide in Cork, Ireland, were identified from coroners’ records and next-of-kin were contacted, as part of the Suicide Support and Information System: A Case-Control Study (SSIS-ACE). Suicide-bereaved family members who participated in SSIS-ACE were also asked for their consent to be contacted for the current study. Twenty-five individuals consented and were contacted via a letter. Nineteen participants agreed to the interview (response rate 76%) but one participant did not consent for the interview to be audio-recorded and was therefore excluded from the qualitative analysis. In one instance, two family members were interviewed together at their request. No one else was present during the interviews and no repeat interviews were conducted. Full details of the recruitment process and reasons for refusal are contained in a previously published mixed methods study (Spillane et al., 2018). Mean time since bereavement during the qualitative interviews was 27.6 months (range: 15–38 months). The sample comprised 11 women and 7 men. Participants were partners (n = 7), parents (n = 5), offspring (n = 4) or siblings (n = 2) of the deceased. Participants were aged between 18–39 years (n = 3), 40–49 years (n = 6), 50–59 years (n = 5) and 60 years and older (n = 4). Most participants were in paid employment (n = 9), with a smaller number being unemployed (n = 4), retired (n = 3) or homemakers (n = 2).

### Data collection

Written informed consent was sought prior to commencement of the interview. Participants’ permission to audio-record the interview was obtained, so all of the interviews were audio-recorded. All of the interviews were conducted by AS, from April 2016–January 2017. AS also analysed all of the interview transcripts. At the time of interview and analysis AS was a PhD student, with a background in population health and health-services research and is experienced in the area of mental health research. AS received specialised training and supervision from an established suicidologist and clinical psychologist to conduct the interviews. Thirteen interviews took place in the participant’s home, two in university research offices and three at a neutral location selected by participants. Facilitating participants to decide the time and location of the interview assisted in putting them at ease and thereby improved the research process. Semi-structured interviews (n = 18) were conducted with the aid of a topic guide in order to explore how family members bereaved by suicide experienced the inquest process. Table I summarises the main questions posed to participants regarding their experiences of the inquest process. This information was collected as part of an interview that was primarily exploring the physical and psychological health impact of the bereavement on family members bereaved by suicide (Spillane et al., 2018). It was explained to participants that the focus of the interview centred on the impact the suicide has had on their psychological and physical health, as well as their subsequent support service needs. Mean length of interviews was 97.5 minutes (Range: 42–180 minutes, SD: 44.2).

**Table I.** Topic guide for exploring participants’ experiences of the inquest process.

Question	Prompts
How long was the inquest since *[deceased’s]* death?	Was this length of time appropriate?
How did you feel in the run up to the inquest?	
Did you attend the inquest?	Why did you/not attend?
How did you find the inquest process?	Positive/negative aspects?
Was there anything about the inquest you particularly liked/disliked?	Timing, location, demeanour of coroner etc.
Did you find any information given at the inquest helpful/unhelpful?	Autopsy results etc.
Did you learn anything new/surprising at the inquest?	Autopsy results etc.
Was the inquest private or were other families there?	How did you feel about that?
	What would you have preferred?
Did the inquest help you to understand what happened around the time of *[deceased’s]* death?	What is clearer/still uncertain?
Can anything be done to make the inquest process easier for family members?	

### Data analysis

Thematic analysis was chosen as the method to analyse the data from the interviews because this is a flexible method that allows for a variety of ontological and epistemological viewpoints (Braun & Clarke, 2006). Thematic analysis represents a systematic framework to code qualitative data in order to identify patterns across the data (Braun & Clarke, 2014). Thematic analysis is especially useful for applied research that focuses on policy and practice or is not completely focused within the field of academia (Braun & Clarke, 2014). There are a number of discrete steps involved in thematic analysis, including familiarising oneself with the data, generating initial codes, searching, reviewing and finally, defining themes (Braun & Clarke, 2006).

The topic guide (Table I) was revised, where appropriate, after interviews to ensure the most pertinent questions were covered throughout the interview process. Field notes and reflections were completed after each interview and formed the basis for the initial analyses. Interview recordings were transcribed verbatim after each interview and initial coding was completed thereafter. Two authors (AS and KMS) coded the data, while each stage of the coding process and development of themes were discussed and reviewed with the research team. Specific consideration was given to discordant cases, whereby the inquest was perceived as positive/mostly positive. NVivo 11 software facilitated the organisation of the data.

## Results

A number of participants found aspects of the inquest process to be inappropriate, insensitive and traumatic. Some were also extremely apprehensive about the process and dreaded the inquest for some time before it occurred. While some described the overall process as “fine”, there were a number of troubling aspects of the inquest that left family members feeling uncomfortable.

Four main themes were identified from the analysis process:

- “Inquest as fearfully unknown”—This main theme relates to participants describing a sense of foreboding or apprehension of the inquest that was largely driven by a lack of information about the inquest and its processes.
- “Structural processes of the inquest”—Participants found some of the structural aspects of the inquest distressing, such as the timing of the inquest and having to hear graphic evidence about the circumstances of the death of their own or someone else’s family member.
- “Enduring public and private pain to obtain answers”—Participants found the public nature of the inquest distressing. Many felt inquests should be private, especially given the very personal nature of information shared about the deceased during the process.
- “Gaining answers and making sense”—Some participants gained clarity about the nature of their family member’s death from the inquest process, which provided a sense of closure to participants.

### Inquest as fearfully unknown

This main theme has one subordinate theme: “Lack of information fuelling heightened emotional reactions”

#### Lack of information fuelling heightened emotional reactions

Participants spoke of a sense of “foreboding” as they waited for the inquest to happen and felt their “life was on hold” until it was over. The inquest was described as “daunting” and “extremely stressful”, with participants on “tenterhooks” until it was over. Being “frightened” and “very nervous” of the inquest was largely driven by a sense of not knowing what the inquest entailed and what form it would take. Some participants were fearful after hearing stories of others’ bad experiences of an inquest, which sometimes led to misinterpretations of what the inquest would entail for them and their family:

I actually had somebody say to me that you’re going to be, they had experienced an inquest and that, I remember she actually kind of frightened me a bit. I was very nervous, she said you’re going to be, it’s like you’re going to be on trial. Like the family can hurl all sorts of questions at you and stuff and my brother was very nervous about that. But, it actually went off grand (P1, partner)

This lack of information and misunderstanding of the inquest process left many family members in a state of distress in the run up to the inquest:

There were things about the inquest that I was completely freaked out about…whereas if I’d had more of an understanding, it was actually fine. It wasn’t any hassle at all like. When I got through it, it was like ‘jeez what was all that about’ (P16, partner)

Family members felt these heightened reactions could have been largely prevented if they had been given practical information about the inquest and what form the process would take:

Then they called me up and they asked a few questions but I had my statement so I was wondering why that wasn’t enough and I thought wouldn’t it be better instead of having this official inquest where they would come in if they had queries about your statement to ask then because ya it was very formal sitting up and swearing and everyone is looking at you and there was a full room the day I went and stuff. You’re never prepared for, they don’t tell you what happens, they don’t prepare you for it (P6, daughter)

Owing to a lack of practical information about the inquest process, some participants were not prepared for the formal aspects of the inquest, such as swearing on the Bible, having to give evidence in a witness box and the courtroom setting. These formalities often reinforced the notion of being “on trial”, which exacerbated grief and guilt reactions in the lead up to the inquest:

I remember being very traumatised because I had to go up to the witness box…I remember I was shaking and I was saying [to deceased] ‘look at what you have me doing now’ and that to me even now was the worst thing [becomes emotional]…the fact that the consequences of her death put me in a witness box to tell legally police and people that didn’t know her, that she had made me do that is what really hit me you know (P3, daughter).

In summary, this theme highlights the distress caused to family members as a result of not knowing what form the inquest would take. Many participants felt fearful of the inquest as a result, which intensified their grief reactions.

### Structural processes of the inquest

This theme relates to some of the formal and structural processes of the inquest, such as the timing of the inquest and having to hear graphic evidence related to their own or someone else’s family member. The timing of the inquest varied for the participants interviewed, with waiting times largely dependent on the presiding coroner and their workload. The majority of participants were passive with regard to the timing of the inquest, but a small minority of participants spoke of actively engaging with the coroner to have the inquest sooner.

Participants often spoke about the timing of the inquest, in relation to the length of time since their family members’ death. Responses were mixed with regard to the most appropriate length of time from the death to inquest. Some felt “the time scale was enough” as “if it had been sooner it would have been a lot worse”. Some described how they needed the time to grieve and come to terms with the death before facing the inquest. One participant appreciated the long wait to the inquest, as it allowed for administrative aspects following the death, such as the deceased’s estate to be settled before the inquest took place. The participant felt they may have had a “different outlook on it if it [inquest] had happened before the estate was settled”. However, others wanted to have the inquest sooner as the family could not get the “suicide note and his clothes” back until the inquest was over. Some had to wait nearly 12 months before their family member’s inquest occurred. One participant spoke about how she was “surprised” her family member’s inquest occurred 4 months after the death as she “didn’t expect it [the inquest] [until] ages after [the death]”. A small number of participants pushed to have the inquest sooner as waiting for 6 months or longer was deemed too long:

The waiting for the inquest, that was [pause]…I think I pushed for it to be sooner, it was in June, but only for I ringing, it would have been after the summer holidays, September [pause] (P1, partner)

Long delays for the inquest also prohibited a small number of participants from attending the inquest:

No I didn’t [attend the inquest] ‘cause that occurred a year later…I had an assignment or project at college due or I had to get some study done, I can’t remember what it was but I didn’t go to the inquest (P8, son)

Some family members found it “very shocking” and “extremely stressful” that they had to listen to graphic evidence regarding the circumstances of their loved one’s death, but also the circumstances of other cases. Specifically, some felt it was “very traumatic” to watch and listen to other families as they were “hysterical” listening to the details of their loved one’s death:

One thing I found very very hard as well was…I had to sit in the room and listen to about 3 or 4 other people and their stories, and before mine had even started I was in tears only listening to other people because some guy died in a car crash, another one committed suicide by [names method]…so you had to sit there and listen to that…before they get to yours…I had to listen to a family that lost a young fella and his girlfriend had been there and she was hysterical listening to the whole thing, I thought that was very traumatic, just watching them, never mind how they were feeling (P6, daughter)

In summary, some family members felt the long wait to the inquest was appropriate as it allowed them time to grieve and to come to terms with the death, while also providing space for administrative aspects of the death, including the deceased’s estate, to be settled. In other instances, family members were dissatisfied with long delays, with a small number pushing to have the inquest sooner.

### Enduring public and private pain to obtain answers

Inquests in Ireland are public and are usually held in a courthouse, but can take place in hotels or local halls. The majority of participants spoke strongly about the very public nature of inquests. A small number of participants described how their family member’s inquest was the last of the day and therefore experienced a private inquest. They felt inquests should be “closed” and only open to family members as it is “a very private thing” and “it is nobody’s business” how the deceased died or the circumstances leading up to the death. One participant described how some of her family members refused to go to the inquest for fear that it would not be private:

They didn’t want to go because they had a fear that it was this public, and it can be public but in our case, it wasn’t private but there was nobody there anyway. [Brother] was very annoyed that this was going to be a public…[Brother] works in [town where inquest was held] and he didn’t want, he had a thing that it was going to be [crowded] like mass but I said nobody knows that it’s [deceased’s] inquest at 10am (P3, daughter)

Because inquests are often scheduled in batches, family members may sit through several inquests before the one relevant to them takes place. Some participants considered themselves fortunate to be the only family members present during the inquest. This was often by chance if their family member’s inquest was the last case scheduled for the day. One participant described how it was “perfect” that “we came in one door and we went out another” so that they were separated from meeting other families, “as we had enough on our own plates”. One family member who had a previous adverse experience at an inquest for a non-suicide death felt strongly opposed to the presence of the media at inquests due to the danger of them publishing sensitive information about their loved one:

I remember the inquest for my *[names family member]*, there was an article in the newspaper afterwards and it said how he died in *[names location of death]*, and I thought it was awful. Nobody needed to know that… (P10, sister)

Other participants spoke about how they would have found it distressing if other families were present, especially when intimate details about their loved one, including the detailing of the deceased’s mental health difficulties and drug or alcohol addiction. One of the primary motivating factors for not wanting other families present was to preserve the intimate details of their loved one and the circumstances surrounding the death. Other participants voiced shame that private aspects of their loved one’s life were laid bare for strangers to hear:

There was another family ahead of us…we heard all of their story which we didn’t need to hear or want to hear…I think it was hard on that family because you could see that they were looking out, going out sideways, not looking at us because they knew that we heard…yeah it was suicide, well it was accidental suicide, she was an alcoholic and she fell down the stairs but the judge gave it out in detail what happened to her…things that we didn’t need to know at all (P14, mother)

This family member was especially nervous that alcohol or drugs could have been involved in her son’s suicide, which she would have found shameful, especially if strangers were to know such details:

I know if it were our case and *[family member]* had fallen down the stairs through drink, I wouldn’t like the family behind me to know all about our private business you know…*[town where deceased lived]* is a small place, we could have known them…I figured we would have kept that *[if alcohol or drugs were involved in suicide]* to ourselves, nobody else’s business, what difference does it make, but as it happened, nothing happened but they went into detail about that woman’s death and her liking for drink and all that (P14, mother)

The fear that family members may have known others at the inquest was not unique and was expressed by two other participants:

If it was someone you knew and you didn’t want them to know the details, you don’t want everyone knowing what goes on either because it’s local…they are hearing about private, what I think are very, very private details. Like as you said if something came out that you didn’t know about, why would you want other people to know? (P6, daughter)

This theme underscores the difficulties experienced by participants when the inquest was not private, but instead was attended by others waiting for their family member’s inquest. Particular difficulties included not wanting others to hear intimate details about their loved one or the circumstances of their death.

### Gaining answers and making sense

This theme has two subordinate themes; “nature of the death and verdict returned” and “learning new information”.

#### Nature of the death and verdict returned

Some family members were unsure of the nature of their loved one’s death prior to the inquest and the inquest often provided answers to their questions. Some participants described how they did not know until the inquest whether alcohol or drugs were involved in their family member’s death. Not knowing the circumstances surrounding the death often troubled family members as it called into question whether the deceased actually intended to take their own life. Others worried that their family member was in pain or may have tried to call for help afterwards. Obtaining clarity on these issues during the inquest often served as a comfort for family members:

When they came out with the autopsy, if you look at the amount of stuff they found in her system, the only consolation was that she went to sleep ‘cause she wasn’t struggling or calling…the key thing was that she wasn’t on the floor trying to find help. In fairness to *[state pathologist]*, she came over and explained this to us and she was really kind of, that this is what happened (P13, father)

Together with the information collected and presented at the inquest this helped some family members to better understand the circumstances of the death and come to a sense of closure regarding the death. In some cases, the evidence gathered indicated the deceased’s careful planning of the suicide, which helped to ease feelings of guilt and blame that they could have prevented the suicide:

Yeah, the inquest was really the final closure on it altogether. There was no doubt about anything, about what the guards [police] found…That was the final bit of closure that said, we’ve taken all the evidence that has been gathered and they were able to lay out the sequence of events, so, for me, for my engineering type mind, it was great to have them laid out and there was absolutely nothing that anyone could have done about it (P4, son)

The inquest also provided most family members with a better understanding of the events that occurred before the death:

She had these panic attack tablets *[names tablet]*, she might have taken one or two over and above, she didn’t know it like that’s what they’ve put it down to. The alcohol in her system was only very small, I think it was only something like half a pint over the limit. In the inquest they said they wouldn’t put it down to that (P2, partner)

Conversely, one family member did not feel that the results of the autopsy mattered to him:

He’s dead. So any extra information about the circumstances wouldn’t make any change to me, I think. It wouldn’t be of any value to me…what would be the difference if he died drunk or not? He’s dead, he’s gone. So, to me there is no difference really (P18, brother)

The majority of family members described how a coroner returned a suicide verdict. An open verdict was given in three cases, which was primarily given due to the lack of physical evidence, such as a suicide note and the specific method chosen. However, in one of the cases, the coroner noted that the deceased did take her own life but he was unable to return such a verdict because of the lack of empirical/physical evidence at the scene of the death. However, in one particular case, some members of the family welcomed the ambiguity of the open verdict as they did not want the stigma of a suicide verdict. These family members were not perturbed that a suicide verdict was not given as “it’s so personal”:

They didn’t give a verdict of suicide, this is what he said, there was no note…there was no physical evidence found…he said that, this poor woman was terrified of her life…and that she took her own life (P3, daughter)

#### Learning new information

For some, the nature of the death and the intent of the deceased was clear. In these cases, “the inquest was more about when the date he died rather than how he died”. For some, the inquest revealed new information about the deceased or circumstances surrounding the death. The autopsy sometimes uncovered physical health problems that the deceased had, which were unknown to them or the family. Hearing such unanticipated news out of the blue was often upsetting for the family:

Here is the stupid thing, at the inquest, *[pathologist]* told me that his heart was twice the size of a normal heart…I said ‘wait a minute, did he have a problem with his heart’ she said ‘yeah, he was a walking time bomb’, in other words, he probably would have died soon anyway…I said to myself, ‘why did you *[kill]* yourself you stupid bastard’, that was my reaction (P9, partner)

The toxicology report often allowed family members an insight into the deceased’s frame of mind. It sometimes removed ambiguity around whether the deceased really intended to take their own life. For some, the presence of toxic levels of alcohol and medication, further reinforced to them that their family member’s death was not an accident. For others, the absence of toxic levels of alcohol and medication, indicated to some family members that they did intend to take their own lives, as they hypothesised that their judgement was not hampered by the presence of toxic levels of substances. Others gained further perspective into their family members’ mental health difficulties as a result of evidence presented at the inquest:

That she was taking her medication and she was still having these bad thoughts, the medication wasn’t working of course. We thought she had given up on her medication (P17, mother)

To summarise, this theme highlights that the inquest can provide family members with additional information about their loved one, including their physical health and any medication or alcohol they had consumed around the time of death. This information can be helpful for some, as it may provide closure regarding the deceased’s intent to take their own life, thereby lessening feelings of guilt and blame.

## Discussion

This qualitative examination found that family members respond to the inquest process in many different ways but often experience the inquest process as traumatic in situations where it is conducted poorly. Additionally, the inquest may be perceived as traumatic if family members are not adequately informed of its purpose and the coronial process generally. Many family members spoke of being extremely apprehensive about the inquest in the months beforehand. This intense fear was driven by not knowing what the inquest would entail and others’ perceptions of being “on trial”. Conversely, some participants interviewed viewed aspects of the inquest as positive. Having a neutral or positive experience of the inquest was sometimes related back to having attended another inquest prior to the deceased’s death and thereby having knowledge about the process. For others, the inquest sometimes provided some clarity with respect to the deceased’s intention to take their own life. Reducing or eliminating family members’ feelings of guilt or blame about the death through the evidence presented at the inquest may be particularly impactful for those closest to the deceased. This is because feelings of guilt and blame can manifest into constant rumination about the death and the deceased, leaving family members unable to move forward and reconstruct their lives after the suicide. Therefore, the inquest process sometimes served as an opportunity for meaning-making about their loved one’s death. Meaning-making is an essential step of reintegration after suicide bereavement, but is especially difficult given the impossibility of knowing the deceased’s frame of mind or motives for taking their own life at the time. This information was often highly valued by family members, contributing to a positive experience surrounding the inquest process.

Although few studies have explored how the inquest process impacts family members bereaved by suicide, the findings corroborate the scant literature on the topic (Biddle, 2003; Chapple et al., 2012), especially with regard to family members being distressed by the timing of the inquest and at hearing graphic evidence during the inquest (Biddle, 2003). A quantitative study noted that over a third of family members encountered problems dealing with the coroner’s office and also being distressed by the media reporting of the inquest (Harwood et al., 2002). Notwithstanding this, a further qualitative study noted that the inquest can sometimes be a positive experience for family members, serving as a source of closure (McKinnon & Chonody, 2014). While this finding was reinforced in this study by a small number of family members, this benefit was outweighed in most cases by the intense anxiety felt before and during the inquest, as well as the other distressing components of the inquest described by family members. Given that this research also aligns with the findings of previous studies conducted in different countries, it further emphasises the credibility, transferability and relevance of this study.

These findings at the individual level highlight a number of key systems level recommendations to improve the inquest process. Recommendations include; (1) the appointment of a liaison officer from the coroner’s office to link in with bereaved family members prior to the inquest to provide comprehensive information on the inquest process and also to proactively facilitate support for the family; (2) consulting with family members regarding the approximate time they deem suitable to hold the inquest, as the timing of the inquest (too soon/delayed etc.) can be potentially distressing for family members; (3) a system should be put in place so that bereaved family members do not have to be present for other inquests while they wait for their own family member’s inquest; (4) the reading of suicide notes or of graphic evidence relating to the death should be restricted, where possible, to reduce distress caused to people attending the inquest; (5) coroners facilitating family members to give a statement prior to the inquest, to relieve them of having to give evidence on the day of the inquest.

While a number of aspects of the inquest were distressing, it is important to highlight some of the positive aspects mentioned by participants. All of the participants that experienced a private inquest, by virtue of them being the last scheduled inquest of the day, found this appropriate and they stated it made the process easier. The compassionate approach of the coroner, the pathologist and members of the police force present were also commended. Participants felt the pleasant demeanour of the coroner was important when they spoke of the factual events that took place around the time of the death. Others praised the coroner and the pathologist who were open to answer any questions or provide further clarification to family members, especially with regard to the toxicology report. Finally, some participants described the compassionate approach of the liaison police officer assigned to them. Participants found it particularly helpful when the liaison officer offered to read out their statement on their behalf. This offer was especially appreciated if the family member was fearful or felt unable to give evidence on the day due to emotional or grief reactions.

### Strengths and limitations

This research has a number of strengths. This study had a representative non-selective sample of suicide-bereaved family members drawn from a pool of all suicides that occurred in Cork, Ireland during the study period. The response rate in this study was high, at 75%. Response rates could not be derived from a number of qualitative studies exploring the inquest process (Biddle, 2003; Chapple et al., 2012) and suicide bereavement more generally (McKinnon & Chonody, 2014; Peters, Cunningham, Murphy, & Jackson, 2016) as recruitment was via sources including, media releases, flyers, websites, newspaper articles, conferences, radio programmes and support organisations. Furthermore, this study achieved a relatively balanced gender distribution (11 women; 7 men), when compared to the existing research on this topic. It is difficult to recruit equal numbers of men and women bereaved by suicide, given that the majority of suicides occur in males, leaving females as the vast majority of people bereaved by suicide. The proportion of males in the current study was 38%, compared to 6% (Biddle, 2003) and 30% (Chapple et al., 2012) in previous research. This increases the opportunity for the voices of males bereaved by the suicide of a spouse, parent or sibling to be heard. The potentially hugely stressful nature of the inquest process, together with the increased suicide risk in males and in people exposed to a family member’s suicide, specifically underlies the importance of capturing the experiences of males bereaved by suicide.

There are some limitations to this research. Additionally, given the lack of standardisation of coroners’ procedures across the country, the experiences of participants in this study may differ to those in another coronial jurisdiction. This point further underlies the importance of standardising coronial procedures not only in Ireland, but in any country that operates within the coronial process.

## Conclusion

The findings from this research illustrate that while some aspects of the inquest were deemed positive, many others were deemed inappropriate and distressing by suicide-bereaved family members. At a time of significant grief and stress, the inquest was a fearful prospect, with some having to wait up to a year after the death. Delayed timing of the inquest, the public nature of the inquest, and hearing graphic evidence were some of the distressing elements of the inquest process. A number of key recommendations arising from this research have been proposed, including having a pre-inquest briefing session with family members outlining the different elements of the inquest and also restricting graphic evidence heard during the inquest. These and the other recommendations proposed are important in order to address distress experienced by suicide-bereaved family members during the inquest process.
